# Markers of NETosis Do Not Predict Neonatal Early Onset Sepsis: A Pilot Study

**DOI:** 10.3389/fped.2019.00555

**Published:** 2020-01-14

**Authors:** Carolin U. Stiel, Chinedu U. Ebenebe, Magdalena Trochimiuk, Laia Pagarols Raluy, Deirdre Vincent, Dominique Singer, Konrad Reinshagen, Michael Boettcher

**Affiliations:** ^1^Department of Pediatric Surgery, University Medical Center Hamburg-Eppendorf, Hamburg, Germany; ^2^Section of Pediatric Intensive Care and Neonatology, Department of Pediatrics, University Medical Center Hamburg-Eppendorf, Hamburg, Germany

**Keywords:** sepsis, neonates, neutrophils, infection, predictor, diagnosis

## Abstract

**Introduction:** Early-onset sepsis in neonates potentially results in substantial morbidity and mortality. A key player in sepsis a neutrophil extracellular traps (NETs) to limit dissemination of pathogens. Aim of this study was to evaluate markers of NET formation in umbilical cord blood as a predictor of neonatal sepsis.

**Methods:** Prospective study including term and preterm neonates. Umbilical cord blood samples were obtained immediately after birth and following markers of inflammation and NET formation were assessed: complete blood count, C-reactive protein (CRP), interleukin 6 (IL-6), levels of cell-free DNA (cfDNA), neutrophil elastase (NE), and myeloperoxidase (MPO). The study population included neonates with confirmed early-onset sepsis and propensity score matched controls.

**Results:** Umbilical cord blood samples of 491 neonates were obtained, of whom 17 neonates (*n* = 17) presented clinical and laboratory signs of infection within the first 72 h postpartum. Seventeen neonates without infection were matched as controls. IL-6 differed significantly between both groups, whereas other infection parameters such as CRP and neutrophil levels, and in particular the NET surrogate markers (cfDNA, NE, MPO), did not show any significant differences.

**Conclusion:** NET markers in umbilical cord blood appear to not predict the onset of neonatal sepsis. These findings probably result from the neonates‘ inability or delayed ability to form NETs, which is suspected to be a main reason for the increased risk of severe infections in neonates, but is also assumed to prevent negative NET-mediated consequences during perinatal adaptation.

## Introduction

Early-onset neonatal sepsis (EOS) remains a life-threatening condition with an incidence of 1.5–3.5/1,000 per live births and a mortality rate ranging from 5 to 10% in developed countries (1, 2). The outcome and prognosis of EOS depends particularly on early and efficient treatment (1). However, clinical signs are non-specific and currently available biological parameters lack sufficient diagnostic accuracy to predict EOS reliably (1, 3). Hence, empirical antibiotics are frequently

administered, resulting in widespread unnecessary exposure to adverse drug effects and nosocomial complications (2). This emphasizes the particular importance of identifying biomarkers with a high degree of accuracy for diagnostics of neonatal sepsis (3).

Neutrophils are considered to be the first line of defense in protecting the body against infection (4) and are the most abundant leukocyte during acute inflammation. In response to infection and injury, neutrophils release DNA filaments that assemble scaffolds known as neutrophil extracellular traps (NETs) (5, 6). NETs are composed of intact, non-fragmented, and double-stranded DNA, which is loaded with histones and enzymes (i.e., myeloperoxidase, neutrophil elastase). Neutrophils release NETs via a programmed cell death pathway (NETosis) or they discharge parts or their nucleus while staying intact (5–8). When released from neutrophils during infection, the proteins and chromatin contained within the NETs form a protective mesh that filters and destroys pathogenic organisms (6). However, NET formation also occurs inappropriately during sterile inflammation resulting in thrombosis, autoimmunity, and tissue damage for instance after ischemia perfusion injuries (9–13).

Recently, markers of NET formation have been shown to correlate with inflammatory diseases like sepsis (14, 15), however, NETs have yet to be evaluated as predictors of sepsis in the neonatal population. Thus, the aim of this study was to evaluate markers of NET formation in umbilical cord blood and to compare their predictive value to current sepsis markers.

## Methods

### Study Design

We conducted a prospective study at the Perinatal Center and the Department of Pediatric Surgery at the University Medical Center Hamburg-Eppendorf (Hamburg, Germany). Our population consisted of term and preterm neonates, who were recruited at birth from March to November 2017. Mother-infant pairs were eligible for enrollment if (a) a cord blood sample was taken immediately after birth, and (b) parental consent was obtained. Neonates with chromosomal abnormalities or major congenital malformations were excluded from the study. Examinations were according to the guidelines of the medical research ethics committee of Hamburg (Ethik-Kommission der Ärztekammer Hamburg, PV5374) and the 1964 Helsinki declaration and its later amendments.

Subjects identified as cases were neonates with confirmed EOS defined as either a positive blood culture or a C-reactive protein (CRP) elevation ≥ 5 mg/l combined with the presence of two or more of the following clinical signs within the first 72 h of life: (1) temperature instability, (2) respiratory symptoms, (3) cardiovascular symptoms (hypotension, tachycardia, bradycardia), (4) neurological symptoms (seizures, hypotonia, lethargy), or (5) abdominal symptoms (vomiting, poor feeding, abdominal distension) (16). A control group was generated by selection of healthy neonates via propensity score matching considering gestational age, birth weight, duration of rupture of membranes (ROM), and the administration of intrapartum antibiotic prophylaxis with a match tolerance of 0.001.

### Sample Collection, Storage, and Analysis

Umbilical cord blood samples were collected immediately after birth. The Institute of Clinical Chemistry and Laboratory Medicine, University Medical Center Hamburg-Eppendorf, performed the laboratory analysis apart from the NET marker evaluation. Following markers were assessed: (1) complete blood count, (2) CRP, (3) interleukin 6 (IL-6). Moreover, levels of cell-free DNA (cfDNA), neutrophil elastase (NE), and myeloperoxidase (MPO), all markers of neutrophil activation and NET formation, were examined (17, 18). CfDNA, as well as NE and MPO, were analyzed as described previously (19).

### Statistics

All data were analyzed with SPSS Statistics 24 (IBM, NY, USA) and GraphPad Prism 8 (GraphPad, CA, USA). As this is a pilot study no formal pre-power study calculation was performed, however, numbers were based on previous trials regarding inflammation and NET formation (20, 21). For all data sets a Mann-Whitney test was applied. Data is shown as median ± interquartile range. The level of significance was set at 0.05.

## Results

During the observational period, parental consents, followed by umbilical cord blood collection, was obtained from 491 neonates. In the study time-frame, 17 neonates presented with clinical and laboratory signs of an infection within the first 72 h and were included in the study based on previously mentioned inclusion criteria. Amongst the 474 infants without clinical signs of infection, 17 neonates were selected for the control group via application of propensity score matching.

Both cases and their matched controls demonstrated similar baseline characteristics: there were no significant differences regarding gestational age [infection 40.14 (37.71–40.29) vs. controls 39.43 (38.86–40.86) weeks, *p* = 0.50], birth weight [infection 3270.00 (2892.50–3920) vs. controls 3445.00 (2990.00–4205) g, *p* = 0.45], and gender distribution (infection 8/17 vs. controls 7/17 female, *p* = 0.73). Moreover, both groups showed similar patterns of perinatal adaptation: 5-min APGAR [infection 9.00 (5.00–10.00) vs. controls 10.00 (09.00–10.00) points, *p* = 0.24] and umbilical PH [infection 7.26 (7.10–7.29) vs. controls 7.18 (7.18–7.25) points, *p* = 0.18]. Maternal age [infection 33.17 (28.43–36.99) vs. controls 31.63 (29.25–35.48) years, *p* = 0.61] was also comparable between both groups. Only one child in the infection, but none in the control group, received steroids to promote fetal lung maturation (*p* = 0.31). Moreover, there were no differences regarding time of ROM before birth [infection 6.38 (0.59–11.15) vs. 6.05 (1.32–11.37) h, *p* = 0.30] and prepartum antibiotic treatment of the mother (infection 9/17 vs. 9/17, *p* = 1.00). There were significant differences regarding amnion-stained amniotic fluid (infection 5/17 vs. 0/17, *p* = 0.017) and maternal temperature [infection 37.50 (37.00–37.90) vs. 36.70 (36.55–37.10) Celsius, *p* = 0.036].

**Figure 1:**
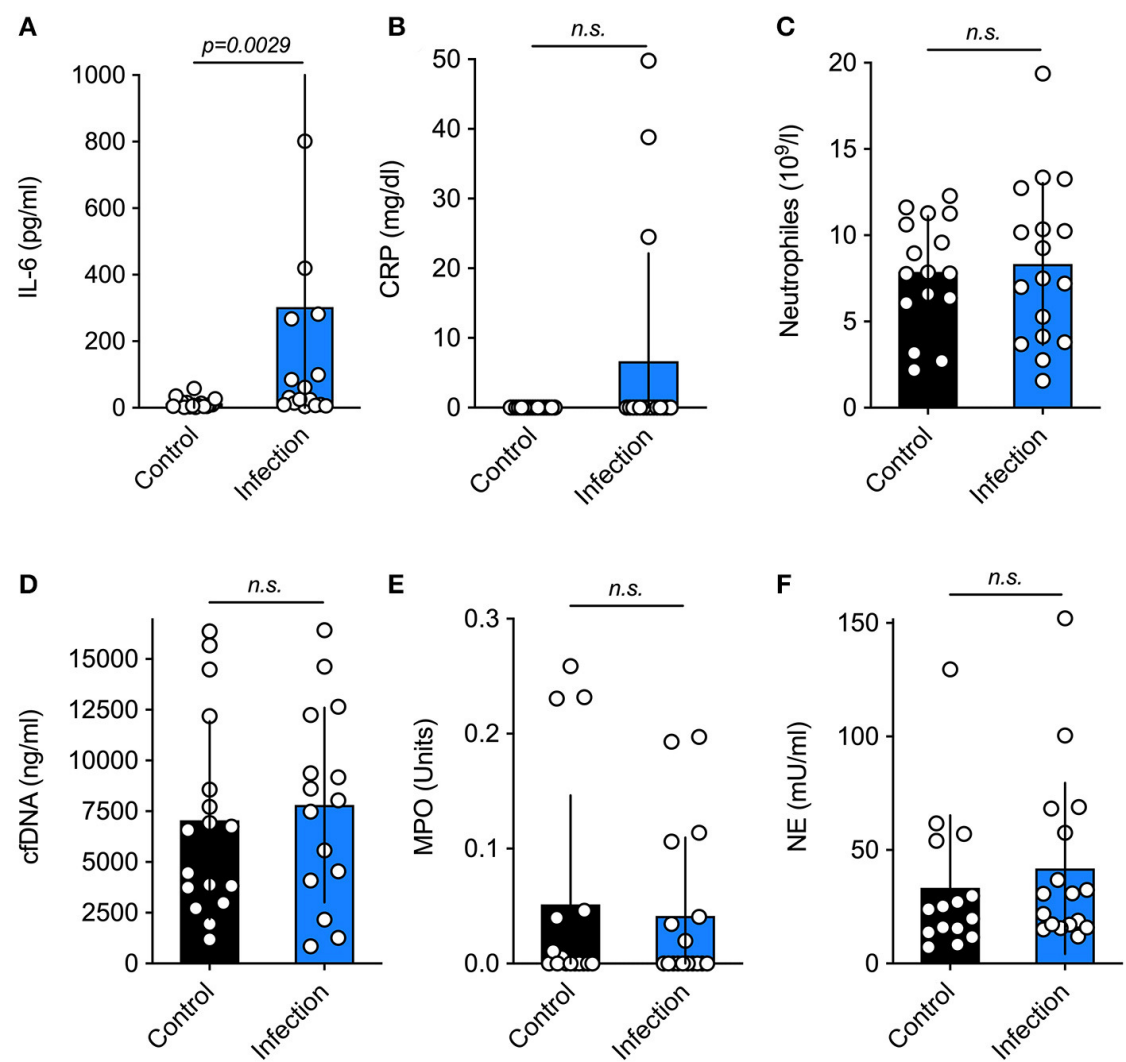
Serum levels of inflammatory markers. Current markers of neonatal infection **(A–C)** and surrogate parameters of NETosis **(D–F)** in the control and the study group. The only significant difference was found for the marker IL-6. Data is shown as Mean ± SD. Statistics: *t*-test or Mann-Whitney test.

There were no differences regarding thrombocyte [infection 278.00 (251.00–301.00) vs. 289.00 (231.99–327.00) 10^9^/l, *p* = 0.57] or leucocyte levels [infection 14.91 (9.52–19.57) vs. 14.68 (10.35–20.43) 10^9^/l, *p* = 0.91] between both groups. With respect to the other parameters, only IL-6 (Figure 1A) differed significantly (*p* = 0.0029) between neonates that developed infections and their matched controls. Other infection parameters, namely CRP (Figure 1B) and neutrophil levels (Figure 1C) were similar in both groups. The *three* subjects with elevated CRP in the umbilical cord blood had amniotic infection syndrome. Moreover, surrogate markers of neutrophil activation and NET formation were also similar in both groups (as shown on Figures 1D–F).

## Discussion

Neonatal sepsis is a common disorder and is associated with high morbidity and mortality while being particularly difficult to predict and thus treat appropriately (2, 22). It has been reported that markers of NET formation, such as cfDNA, are excellent biomarkers of sepsis in adult polytrauma patients (20). However, to date no study has evaluated markers of NET formation for prediction of sepsis in the neonatal population. Thus, as the collection of umbilical cord blood is easy and non-invasive, the aim of this current study was to evaluate NET markers in umbilical cord blood as predictors of EOS. However, no differences of the NET markers were found between neonates that developed an infection within 72 h postpartum and their matched controls.

The chief reason for the similar concentrations of surrogate markers of neutrophil activation and NET formation amongst cases and controls is the inability of neonates to form NETs. Yost et al. reported that neutrophils of both premature and term born infants have very low NE activity and fail to form NETs in response to inflammatory stimulation i.e., with LPS. This impairment of NET formation was shown to be accompanied by a deficiency in extracellular bacterial killing, whereas the amount of bacterial killing due to phagocytosis by neutrophils was not affected (21). It has been speculated that these quantitative and functional deficiencies of neonatal neutrophils are the main cause for the elevated risks of neonatal infections (23). Yost et al. also found that the impairment of neonatal neutrophils is due to a NET inhibitory factor (nNIF) that is expressed during the first days of life. In fact, Yost et al. suggest a tight control of perinatal NET formation to prevent negative repercussions during perinatal adaptation, such as hyperinflammation, NET-mediated vascular injury, and thrombosis (24).

Recent studies evaluating neonatal, and not umbilical cord blood, surrogate markers of NET formation showed an association with sepsis (25, 26): elevated circulating cfDNA levels in neonatal plasma have been associated with late-onset sepsis, as well as necrotizing enterocolitis, diagnosis (25). Moreover, neutrophils capable of releasing NETs have also been described as potential sepsis biomarkers in neonates (11, 26).

In this study, no significant difference in neutrophil levels was found between the cases and controls. However, when analyzing the entire study population (*n* = 491) neutrophil counts were significantly higher in neonates with EOS (*p* < 0.001) (unpublished data). Similarly, although no significant differences of the NET markers were found between the two groups in this study, there appeared to be a trend of elevated cfDNA and NE in neonates with infection compared to those without infection. Hence, as this was a pilot study with a limited number of subjects, one might argue that differences between groups would be more pronounced and potentially even significant in a larger study group.

Overall the main strengths of the current study are the propensity score match, the utilization of umbilical cord blood with a prospective study design, and the utilization of established methods of analyzing different NET markers.

In conclusion, we did not find a significant correlation between NET markers in umbilical cord blood and neonatal infection within 72 h postpartum. Whether this is due to the neonates' limited capacity to form NETs or the size of our study population remains to be seen. However, NET markers should not be dismissed as potential biomarkers of neonatal infection, as NET formation seems to normalize after day three postpartum (24). Thus, further studies should include larger populations of newborns to evaluate umbilical cord blood in the backdrop of EOS and older neonates to specify the different role of NET marker in EOS and late onset sepsis.

## Data Availability Statement

All datasets generated for this study are included in the article/supplementary material.

## Ethics Statement

The studies involving human participants were reviewed and approved by Ethik-Kommission der Ärztekammer Hamburg, PV5374. Written informed consent to participate in this study was provided by the participants' legal guardian/next of kin.

## Author Contributions

CS, CE, and LP acquired the data, drafted the initial manuscript, and approved the final manuscript as submitted. MT acquired the data and approved the final manuscript as submitted. DV, DS, and KR conceptualized and designed the study, and approved the final manuscript as submitted. MB conceptualized and designed the study, acquired the data, performed statistics, drafted the initial manuscript, and approved the final manuscript as submitted. All authors approved the final manuscript as submitted and agree to be accountable for all aspects of the work.

### Conflict of Interest

The authors declare that the research was conducted in the absence of any commercial or financial relationships that could be construed as a potential conflict of interest.

## Abbreviations

NETs: neutrophil extracellular traps
cfDNA: cell-free DNA
NE: neutrophil elastase
MPO: myeloperoxidase
CRP: C-reactive protein (CRP)
IL-6: interleukin 6.
